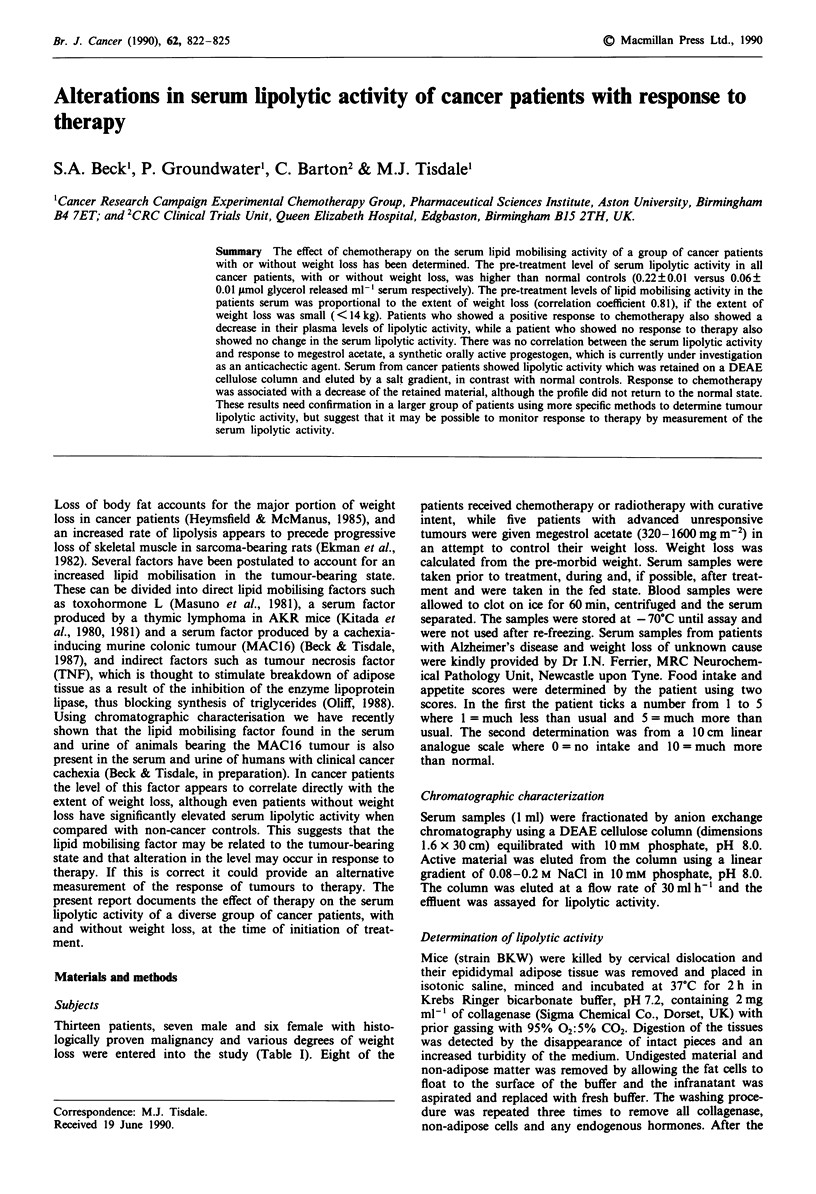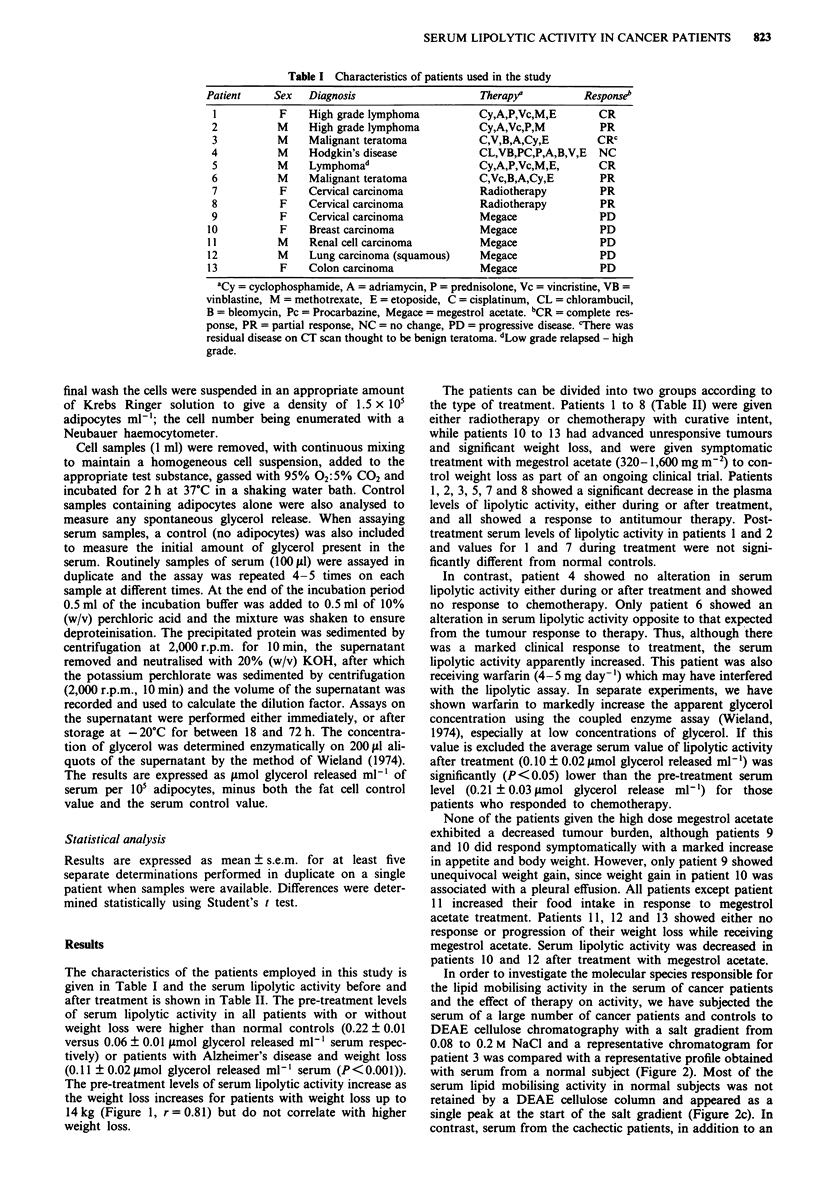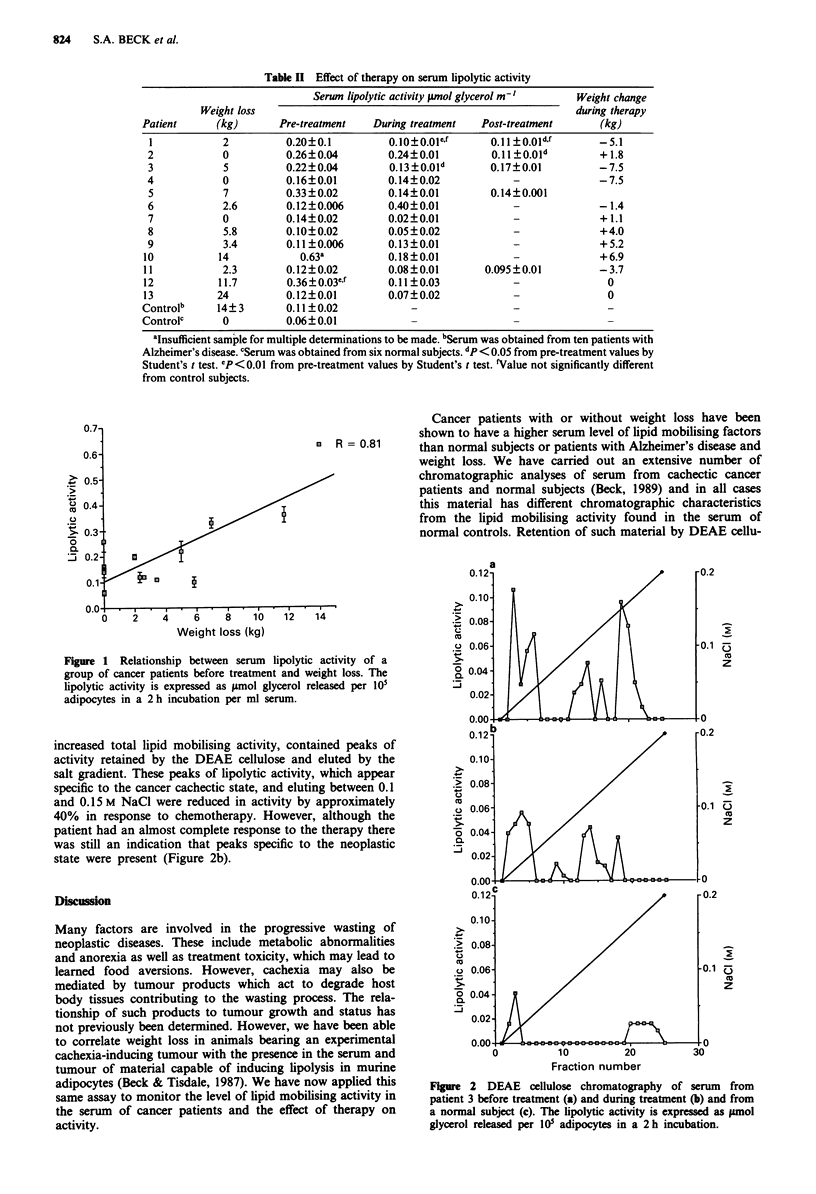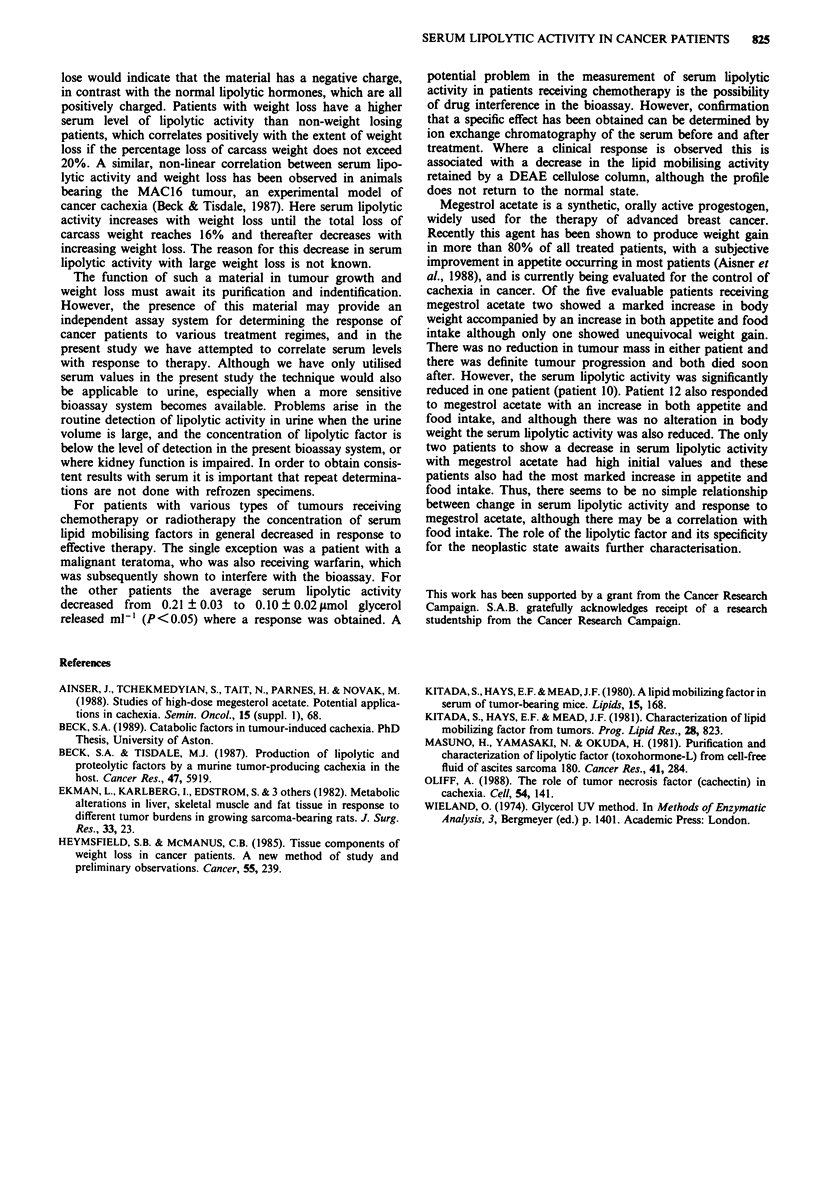# Alterations in serum lipolytic activity of cancer patients with response to therapy.

**DOI:** 10.1038/bjc.1990.385

**Published:** 1990-11

**Authors:** S. A. Beck, P. Groundwater, C. Barton, M. J. Tisdale

**Affiliations:** Cancer Research Campaign Experimental Chemotherapy Group, Pharmaceutical Sciences Institute, Aston University, Birmingham.

## Abstract

The effect of chemotherapy on the serum lipid mobilising activity of a group of cancer patients with or without weight loss has been determined. The pre-treatment level of serum lipolytic activity in all cancer patients, with or without weight loss, was higher than normal controls (0.22 +/- 0.01 versus 0.06 +/- 0.01 mumols glycerol released ml-1 serum respectively). The pre-treatment levels of lipid mobilising activity in the patients serum was proportional to the extent of weight loss (correlation coefficient 0.81), if the extent of weight loss was small (less than 14 kg). Patients who showed a positive response to chemotherapy also showed a decrease in their plasma levels of lipolytic activity, while a patient who showed no response to therapy also showed no change in the serum lipolytic activity. There was no correlation between the serum lipolytic activity and response to megestrol acetate, a synthetic orally active progestogen, which is currently under investigation as an anticachectic agent. Serum from cancer patients showed lipolytic activity which was retained on a DEAE cellulose column and eluted by a salt gradient, in contrast with normal controls. Response to chemotherapy was associated with a decrease of the retained material, although the profile did not return to the normal state. These results need confirmation in a larger group of patients using more specific methods to determine tumour lipolytic activity, but suggest that it may be possible to monitor response to therapy by measurement of the serum lipolytic activity.


Br. J. Cancer (1990), 62, 822–825 © Macmillan Press Ltd., 1990

### Alterations in serum lipolytic activity of cancer patients with response to therapy

S.A. Beck[1], P. Groundwater[1], C. Barton[2] & M.J. Tisdale[1]

[1]*Cancer Research Campaign Experimental Chemotherapy Group, Pharmaceutical Sciences Institute, Aston University, Birmingham B4 7ET; and* [2]*CRC Clinical Trials Unit, Queen Elizabeth Hospital, Edgbaston, Birmingham B15 2TH, UK.*

**Summary** The effect of chemotherapy on the serum lipid mobilising activity of a group of cancer patients with or without weight loss has been determined. The pre-treatment level of serum lipolytic activity in all cancer patients, with or without weight loss, was higher than normal controls ($0.22 \pm 0.01$ versus $0.06 \pm 0.01$ μmol glycerol released $ml^{-1}$ serum respectively). The pre-treatment levels of lipid mobilising activity in the patients serum was proportional to the extent of weight loss (correlation coefficient 0.81), if the extent of weight loss was small ($< 14$ kg). Patients who showed a positive response to chemotherapy also showed a decrease in their plasma levels of lipolytic activity, while a patient who showed no response to therapy also showed no change in the serum lipolytic activity. There was no correlation between the serum lipolytic activity and response to megestrol acetate, a synthetic orally active progestogen, which is currently under investigation as an anticachectic agent. Serum from cancer patients showed lipolytic activity which was retained on a DEAE cellulose column and eluted by a salt gradient, in contrast with normal controls. Response to chemotherapy was associated with a decrease of the retained material, although the profile did not return to the normal state. These results need confirmation in a larger group of patients using more specific methods to determine tumour lipolytic activity, but suggest that it may be possible to monitor response to therapy by measurement of the serum lipolytic activity.

Loss of body fat accounts for the major portion of weight loss in cancer patients (Heymsfield & McManus, 1985), and an increased rate of lipolysis appears to precede progressive loss of skeletal muscle in sarcoma-bearing rats (Ekman *et al.*, 1982). Several factors have been postulated to account for an increased lipid mobilisation in the tumour-bearing state. These can be divided into direct lipid mobilising factors such as toxohormone L (Masuno *et al.*, 1981), a serum factor produced by a thymic lymphoma in AKR mice (Kitada *et al.*, 1980, 1981) and a serum factor produced by a cachexia-inducing murine colonic tumour (MAC16) (Beck & Tisdale, 1987), and indirect factors such as tumour necrosis factor (TNF), which is thought to stimulate breakdown of adipose tissue as a result of the inhibition of the enzyme lipoprotein lipase, thus blocking synthesis of triglycerides (Oliff, 1988). Using chromatographic characterisation we have recently shown that the lipid mobilising factor found in the serum and urine of animals bearing the MAC16 tumour is also present in the serum and urine of humans with clinical cancer cachexia (Beck & Tisdale, in preparation). In cancer patients the level of this factor appears to correlate directly with the extent of weight loss, although even patients without weight loss have significantly elevated serum lipolytic activity when compared with non-cancer controls. This suggests that the lipid mobilising factor may be related to the tumour-bearing state and that alteration in the level may occur in response to therapy. If this is correct it could provide an alternative measurement of the response of tumours to therapy. The present report documents the effect of therapy on the serum lipolytic activity of a diverse group of cancer patients, with and without weight loss, at the time of initiation of treatment.

### Materials and methods

### Subjects

Thirteen patients, seven male and six female with histologically proven malignancy and various degrees of weight loss were entered into the study (Table I). Eight of the patients received chemotherapy or radiotherapy with curative intent, while five patients with advanced unresponsive tumours were given megestrol acetate (320–1600 mg $m^{-2}$) in an attempt to control their weight loss. Weight loss was calculated from the pre-morbid weight. Serum samples were taken prior to treatment, during and, if possible, after treatment and were taken in the fed state. Blood samples were allowed to clot on ice for 60 min, centrifuged and the serum separated. The samples were stored at $-70°C$ until assay and were not used after re-freezing. Serum samples from patients with Alzheimer's disease and weight loss of unknown cause were kindly provided by Dr I.N. Ferrier, MRC Neurochemical Pathology Unit, Newcastle upon Tyne. Food intake and appetite scores were determined by the patient using two scores. In the first the patient ticks a number from 1 to 5 where 1 = much less than usual and 5 = much more than usual. The second determination was from a 10 cm linear analogue scale where 0 = no intake and 10 = much more than normal.

### Chromatographic characterization

Serum samples (1 ml) were fractionated by anion exchange chromatography using a DEAE cellulose column (dimensions 1.6 × 30 cm) equilibrated with 10 mM phosphate, pH 8.0. Active material was eluted from the column using a linear gradient of 0.08–0.2 M NaCl in 10 mM phosphate, pH 8.0. The column was eluted at a flow rate of 30 ml $h^{-1}$ and the effluent was assayed for lipolytic activity.

### Determination of lipolytic activity

Mice (strain BKW) were killed by cervical dislocation and their epididymal adipose tissue was removed and placed in isotonic saline, minced and incubated at 37°C for 2 h in Krebs Ringer bicarbonate buffer, pH 7.2, containing 2 mg $ml^{-1}$ of collagenase (Sigma Chemical Co., Dorset, UK) with prior gassing with 95% $O_2$:5% $CO_2$. Digestion of the tissues was detected by the disappearance of intact pieces and an increased turbidity of the medium. Undigested material and non-adipose matter was removed by allowing the fat cells to float to the surface of the buffer and the infranatant was aspirated and replaced with fresh buffer. The washing procedure was repeated three times to remove all collagenase, non-adipose cells and any endogenous hormones. After the

Correspondence: M.J. Tisdale.
Received 19 June 1990.

**Table I** Characteristics of patients used in the study

| *Patient* | *Sex* | *Diagnosis* | *Therapy*[a] | *Response*[b] |
|---|---|---|---|---|
| 1 | F | High grade lymphoma | Cy,A,P,Vc,M,E | CR |
| 2 | M | High grade lymphoma | Cy,A,Vc,P,M | PR |
| 3 | M | Malignant teratoma | C,V,B,A,Cy,E | CR[c] |
| 4 | M | Hodgkin's disease | CL,VB,PC,P,A,B,V,E | NC |
| 5 | M | Lymphoma[d] | Cy,A,P,Vc,M,E, | CR |
| 6 | M | Malignant teratoma | C,Vc,B,A,Cy,E | PR |
| 7 | F | Cervical carcinoma | Radiotherapy | PR |
| 8 | F | Cervical carcinoma | Radiotherapy | PR |
| 9 | F | Cervical carcinoma | Megace | PD |
| 10 | F | Breast carcinoma | Megace | PD |
| 11 | M | Renal cell carcinoma | Megace | PD |
| 12 | M | Lung carcinoma (squamous) | Megace | PD |
| 13 | F | Colon carcinoma | Megace | PD |

[a]Cy = cyclophosphamide, A = adriamycin, P = prednisolone, Vc = vincristine, VB = vinblastine, M = methotrexate, E = etoposide, C = cisplatinum, CL = chlorambucil, B = bleomycin, Pc = Procarbazine, Megace = megestrol acetate. [b]CR = complete response, PR = partial response, NC = no change, PD = progressive disease. [c]There was residual disease on CT scan thought to be benign teratoma. [d]Low grade relapsed – high grade.

final wash the cells were suspended in an appropriate amount of Krebs Ringer solution to give a density of $1.5 \times 10^5$ adipocytes $ml^{-1}$; the cell number being enumerated with a Neubauer haemocytometer.

Cell samples (1 ml) were removed, with continuous mixing to maintain a homogeneous cell suspension, added to the appropriate test substance, gassed with 95% $O_2$:5% $CO_2$ and incubated for 2 h at 37°C in a shaking water bath. Control samples containing adipocytes alone were also analysed to measure any spontaneous glycerol release. When assaying serum samples, a control (no adipocytes) was also included to measure the initial amount of glycerol present in the serum. Routinely samples of serum (100 µl) were assayed in duplicate and the assay was repeated 4–5 times on each sample at different times. At the end of the incubation period 0.5 ml of the incubation buffer was added to 0.5 ml of 10% (w/v) perchloric acid and the mixture was shaken to ensure deproteinisation. The precipitated protein was sedimented by centrifugation at 2,000 r.p.m. for 10 min, the supernatant removed and neutralised with 20% (w/v) KOH, after which the potassium perchlorate was sedimented by centrifugation (2,000 r.p.m., 10 min) and the volume of the supernatant was recorded and used to calculate the dilution factor. Assays on the supernatant were performed either immediately, or after storage at −20°C for between 18 and 72 h. The concentration of glycerol was determined enzymatically on 200 µl aliquots of the supernatant by the method of Wieland (1974). The results are expressed as µmol glycerol released $ml^{-1}$ of serum per $10^5$ adipocytes, minus both the fat cell control value and the serum control value.

*Statistical analysis*

Results are expressed as mean ± s.e.m. for at least five separate determinations performed in duplicate on a single patient when samples were available. Differences were determined statistically using Student's $t$ test.

### Results

The characteristics of the patients employed in this study is given in Table I and the serum lipolytic activity before and after treatment is shown in Table II. The pre-treatment levels of serum lipolytic activity in all patients with or without weight loss were higher than normal controls (0.22 ± 0.01 versus 0.06 ± 0.01 µmol glycerol released $ml^{-1}$ serum respectively) or patients with Alzheimer's disease and weight loss (0.11 ± 0.02 µmol glycerol released $ml^{-1}$ serum ($P < 0.001$)). The pre-treatment levels of serum lipolytic activity increase as the weight loss increases for patients with weight loss up to 14 kg (Figure 1, $r = 0.81$) but do not correlate with higher weight loss.

The patients can be divided into two groups according to the type of treatment. Patients 1 to 8 (Table II) were given either radiotherapy or chemotherapy with curative intent, while patients 10 to 13 had advanced unresponsive tumours and significant weight loss, and were given symptomatic treatment with megestrol acetate (320–1,600 mg $m^{-2}$) to control weight loss as part of an ongoing clinical trial. Patients 1, 2, 3, 5, 7 and 8 showed a significant decrease in the plasma levels of lipolytic activity, either during or after treatment, and all showed a response to antitumour therapy. Post-treatment serum levels of lipolytic activity in patients 1 and 2 and values for 1 and 7 during treatment were not significantly different from normal controls.

In contrast, patient 4 showed no alteration in serum lipolytic activity either during or after treatment and showed no response to chemotherapy. Only patient 6 showed an alteration in serum lipolytic activity opposite to that expected from the tumour response to therapy. Thus, although there was a marked clinical response to treatment, the serum lipolytic activity apparently increased. This patient was also receiving warfarin (4–5 mg $day^{-1}$) which may have interfered with the lipolytic assay. In separate experiments, we have shown warfarin to markedly increase the apparent glycerol concentration using the coupled enzyme assay (Wieland, 1974), especially at low concentrations of glycerol. If this value is excluded the average serum value of lipolytic activity after treatment (0.10 ± 0.02 µmol glycerol released $ml^{-1}$) was significantly ($P < 0.05$) lower than the pre-treatment serum level (0.21 ± 0.03 µmol glycerol release $ml^{-1}$) for those patients who responded to chemotherapy.

None of the patients given the high dose megestrol acetate exhibited a decreased tumour burden, although patients 9 and 10 did respond symptomatically with a marked increase in appetite and body weight. However, only patient 9 showed unequivocal weight gain, since weight gain in patient 10 was associated with a pleural effusion. All patients except patient 11 increased their food intake in response to megestrol acetate treatment. Patients 11, 12 and 13 showed either no response or progression of their weight loss while receiving megestrol acetate. Serum lipolytic activity was decreased in patients 10 and 12 after treatment with megestrol acetate.

In order to investigate the molecular species responsible for the lipid mobilising activity in the serum of cancer patients and the effect of therapy on activity, we have subjected the serum of a large number of cancer patients and controls to DEAE cellulose chromatography with a salt gradient from 0.08 to 0.2 M NaCl and a representative chromatogram for patient 3 was compared with a representative profile obtained with serum from a normal subject (Figure 2). Most of the serum lipid mobilising activity in normal subjects was not retained by a DEAE cellulose column and appeared as a single peak at the start of the salt gradient (Figure 2c). In contrast, serum from the cachectic patients, in addition to an

**Table II** Effect of therapy on serum lipolytic activity

| Patient | Weight loss (kg) | Serum lipolytic activity μmol glycerol $m^{-1}$ Pre-treatment | During treatment | Post-treatment | Weight change during therapy (kg) |
|---|---|---|---|---|---|
| 1 | 2 | 0.20±0.1 | 0.10±0.01[e,f] | 0.11±0.01[d,f] | −5.1 |
| 2 | 0 | 0.26±0.04 | 0.24±0.01 | 0.11±0.01[d] | +1.8 |
| 3 | 5 | 0.22±0.04 | 0.13±0.01[d] | 0.17±0.01 | −7.5 |
| 4 | 0 | 0.16±0.01 | 0.14±0.02 | – | −7.5 |
| 5 | 7 | 0.33±0.02 | 0.14±0.01 | 0.14±0.001 | |
| 6 | 2.6 | 0.12±0.006 | 0.40±0.01 | – | −1.4 |
| 7 | 0 | 0.14±0.02 | 0.02±0.01 | – | +1.1 |
| 8 | 5.8 | 0.10±0.02 | 0.05±0.02 | – | +4.0 |
| 9 | 3.4 | 0.11±0.006 | 0.13±0.01 | – | +5.2 |
| 10 | 14 | 0.63[a] | 0.18±0.01 | – | +6.9 |
| 11 | 2.3 | 0.12±0.02 | 0.08±0.01 | 0.095±0.01 | −3.7 |
| 12 | 11.7 | 0.36±0.03[e,f] | 0.11±0.03 | – | 0 |
| 13 | 24 | 0.12±0.01 | 0.07±0.02 | – | 0 |
| Control[b] | 14±3 | 0.11±0.02 | – | – | – |
| Control[c] | 0 | 0.06±0.01 | – | – | – |

[a]Insufficient sample for multiple determinations to be made. [b]Serum was obtained from ten patients with Alzheimer's disease. [c]Serum was obtained from six normal subjects. [d]$P<0.05$ from pre-treatment values by Student's $t$ test. [e]$P<0.01$ from pre-treatment values by Student's $t$ test. [f]Value not significantly different from control subjects.

**Figure 1** Relationship between serum lipolytic activity of a group of cancer patients before treatment and weight loss. The lipolytic activity is expressed as μmol glycerol released per $10^5$ adipocytes in a 2 h incubation per ml serum.

increased total lipid mobilising activity, contained peaks of activity retained by the DEAE cellulose and eluted by the salt gradient. These peaks of lipolytic activity, which appear specific to the cancer cachectic state, and eluting between 0.1 and 0.15 M NaCl were reduced in activity by approximately 40% in response to chemotherapy. However, although the patient had an almost complete response to the therapy there was still an indication that peaks specific to the neoplastic state were present (Figure 2b).

### Discussion

Many factors are involved in the progressive wasting of neoplastic diseases. These include metabolic abnormalities and anorexia as well as treatment toxicity, which may lead to learned food aversions. However, cachexia may also be mediated by tumour products which act to degrade host body tissues contributing to the wasting process. The relationship of such products to tumour growth and status has not previously been determined. However, we have been able to correlate weight loss in animals bearing an experimental cachexia-inducing tumour with the presence in the serum and tumour of material capable of inducing lipolysis in murine adipocytes (Beck & Tisdale, 1987). We have now applied this same assay to monitor the level of lipid mobilising activity in the serum of cancer patients and the effect of therapy on activity.

Cancer patients with or without weight loss have been shown to have a higher serum level of lipid mobilising factors than normal subjects or patients with Alzheimer's disease and weight loss. We have carried out an extensive number of chromatographic analyses of serum from cachectic cancer patients and normal subjects (Beck, 1989) and in all cases this material has different chromatographic characteristics from the lipid mobilising activity found in the serum of normal controls. Retention of such material by DEAE cellu-

**Figure 2** DEAE cellulose chromatography of serum from patient 3 before treatment (**a**) and during treatment (**b**) and from a normal subject (**c**). The lipolytic activity is expressed as μmol glycerol released per $10^5$ adipocytes in a 2 h incubation.

lose would indicate that the material has a negative charge, in contrast with the normal lipolytic hormones, which are all positively charged. Patients with weight loss have a higher serum level of lipolytic activity than non-weight losing patients, which correlates positively with the extent of weight loss if the percentage loss of carcass weight does not exceed 20%. A similar, non-linear correlation between serum lipolytic activity and weight loss has been observed in animals bearing the MAC16 tumour, an experimental model of cancer cachexia (Beck & Tisdale, 1987). Here serum lipolytic activity increases with weight loss until the total loss of carcass weight reaches 16% and thereafter decreases with increasing weight loss. The reason for this decrease in serum lipolytic activity with large weight loss is not known.

The function of such a material in tumour growth and weight loss must await its purification and indentification. However, the presence of this material may provide an independent assay system for determining the response of cancer patients to various treatment regimes, and in the present study we have attempted to correlate serum levels with response to therapy. Although we have only utilised serum values in the present study the technique would also be applicable to urine, especially when a more sensitive bioassay system becomes available. Problems arise in the routine detection of lipolytic activity in urine when the urine volume is large, and the concentration of lipolytic factor is below the level of detection in the present bioassay system, or where kidney function is impaired. In order to obtain consistent results with serum it is important that repeat determinations are not done with refrozen specimens.

For patients with various types of tumours receiving chemotherapy or radiotherapy the concentration of serum lipid mobilising factors in general decreased in response to effective therapy. The single exception was a patient with a malignant teratoma, who was also receiving warfarin, which was subsequently shown to interfere with the bioassay. For the other patients the average serum lipolytic activity decreased from $0.21 \pm 0.03$ to $0.10 \pm 0.02\,\mu$mol glycerol released ml$^{-1}$ ($P < 0.05$) where a response was obtained. A potential problem in the measurement of serum lipolytic activity in patients receiving chemotherapy is the possibility of drug interference in the bioassay. However, confirmation that a specific effect has been obtained can be determined by ion exchange chromatography of the serum before and after treatment. Where a clinical response is observed this is associated with a decrease in the lipid mobilising activity retained by a DEAE cellulose column, although the profile does not return to the normal state.

Megestrol acetate is a synthetic, orally active progestogen, widely used for the therapy of advanced breast cancer. Recently this agent has been shown to produce weight gain in more than 80% of all treated patients, with a subjective improvement in appetite occurring in most patients (Aisner *et al*., 1988), and is currently being evaluated for the control of cachexia in cancer. Of the five evaluable patients receiving megestrol acetate two showed a marked increase in body weight accompanied by an increase in both appetite and food intake although only one showed unequivocal weight gain. There was no reduction in tumour mass in either patient and there was definite tumour progression and both died soon after. However, the serum lipolytic activity was significantly reduced in one patient (patient 10). Patient 12 also responded to megestrol acetate with an increase in both appetite and food intake, and although there was no alteration in body weight the serum lipolytic activity was also reduced. The only two patients to show a decrease in serum lipolytic activity with megestrol acetate had high initial values and these patients also had the most marked increase in appetite and food intake. Thus, there seems to be no simple relationship between change in serum lipolytic activity and response to megestrol acetate, although there may be a correlation with food intake. The role of the lipolytic factor and its specificity for the neoplastic state awaits further characterisation.

This work has been supported by a grant from the Cancer Research Campaign. S.A.B. gratefully acknowledges receipt of a research studentship from the Cancer Research Campaign.

### References

AINSER, J., TCHEKMEDYIAN, S., TAIT, N., PARNES, H. & NOVAK, M. (1988). Studies of high-dose megesterol acetate. Potential applications in cachexia. *Semin. Oncol.*, **15** (suppl. 1), 68.

BECK, S.A. (1989). Catabolic factors in tumour-induced cachexia. PhD Thesis, University of Aston.

BECK, S.A. & TISDALE, M.J. (1987). Production of lipolytic and proteolytic factors by a murine tumor-producing cachexia in the host. *Cancer Res.*, **47**, 5919.

EKMAN, L., KARLBERG, I., EDSTROM, S. & 3 others (1982). Metabolic alterations in liver, skeletal muscle and fat tissue in response to different tumor burdens in growing sarcoma-bearing rats. *J. Surg. Res.*, **33**, 23.

HEYMSFIELD, S.B. & MCMANUS, C.B. (1985). Tissue components of weight loss in cancer patients. A new method of study and preliminary observations. *Cancer*, **55**, 239.

KITADA, S., HAYS, E.F. & MEAD, J.F. (1980). A lipid mobilizing factor in serum of tumor-bearing mice. *Lipids*, **15**, 168.

KITADA, S., HAYS, E.F. & MEAD, J.F. (1981). Characterization of lipid mobilizing factor from tumors. *Prog. Lipid Res.*, **28**, 823.

MASUNO, H., YAMASAKI, N. & OKUDA, H. (1981). Purification and characterization of lipolytic factor (toxohormone-L) from cell-free fluid of ascites sarcoma 180. *Cancer Res.*, **41**, 284.

OLIFF, A. (1988). The role of tumor necrosis factor (cachectin) in cachexia. *Cell*, **54**, 141.

WIELAND, O. (1974). Glycerol UV method. In *Methods of Enzymatic Analysis, 3*, Bergmeyer (ed.) p. 1401. Academic Press: London.